# Analysis of circulating angiogenic biomarkers from patients in two phase III trials in lung cancer of chemotherapy alone or chemotherapy and thalidomide

**DOI:** 10.1038/bjc.2012.50

**Published:** 2012-02-21

**Authors:** R J Young, A W Tin, N J Brown, M Jitlal, S M Lee, P J Woll

**Affiliations:** 1 Academic Unit of Clinical Oncology, School of Medicine & Biomedical Sciences, University of Sheffield, Sheffield S10 2RX, UK; 2 Academic Unit of Surgical Oncology, University of Sheffield, Sheffield, UK; 3 Cancer Research UK & University College London Cancer Trials Centre, London, UK; 4 UCL Cancer Institute, University College London, London, UK

**Keywords:** lung cancer, thalidomide, angiogenesis, biomarker

## Abstract

**Background:**

Thalidomide has potent anti-inflammatory and anti-angiogenic properties. It was evaluated in combination with chemotherapy in two randomised placebo-controlled trials in patients with small cell lung cancer (SCLC, *n*=724) and advanced non-small cell lung cancer (NSCLC, *n*=722). Neither study demonstrated an improvement in overall survival with the addition of thalidomide to chemotherapy. This study investigated circulating angiogenic biomarkers in a subset of these patients.

**Methods:**

Serial plasma samples were collected in a cohort of patients enrolled in these two trials (*n*=95). Vascular endothelial growth factor (VEGF), soluble truncated form of VEGF receptor-2 (sVEGFR-2), interleukin-8 (IL-8), tumour necrosis factor-*α* (TNF-*α*), basic fibroblast growth factor (bFGF) and soluble intercellular adhesion molecule-1 (sICAM-1) levels were measured by enzyme-linked immunosorbent assays. Results were correlated with patient clinical data including stage, response rate and progression-free survival (PFS).

**Results:**

Baseline biomarker levels were not significantly different between SCLC and NSCLC. For pooled treatment groups, limited stage SCLC was associated with lower baseline VEGF (*P*=0.046), sICAM-1 (*P*=0.008) and IL-8 (*P*=0.070) than extensive stage disease. Low baseline IL-8 was associated with a significantly improved PFS in both SCLC and NSCLC (*P*=0.028), and a greater reduction in IL-8 was associated with a significantly improved tumour response (*P*=0.035). Baseline angiogenic factor levels, however, did not predict response to thalidomide.

**Conclusion:**

Circulating angiogenic biomarkers did not identify patients who benefited from thalidomide treatment.

Angiogenesis is a critical step in tumour growth, and the development of angiogenic-targeted therapy is the focus of intense research. High levels of vascular endothelial growth factor (VEGF) and tumour vessel density are poor prognostic markers in both small cell lung cancer (SCLC) and non-small cell lung cancer (NSCLC) (Herbst *et al*, 2005). VEGF is a key regulator of angiogenesis and the humanised VEGF monoclonal antibody bevacizumab has been shown to prolong survival when given in combination with chemotherapy in NSCLC (Sandler *et al*, 2006).

Thalidomide is a synthetic glutamic acid derivative, with immunomodulatory and anti-proliferative activity with proven clinical activity in multiple myeloma and myelodysplastic syndromes (Melchert and List, 2007). It inhibits tumour necrosis factor-*α* (TNF-*α*) production by increasing degradation of TNF-*α* mRNA. It inhibits nuclear factor kappa B, a key regulator of TNF-*α* and interleukin-8 (IL-8) production, and reduces the expression of cell surface markers such as soluble intercellular adhesion molecule-1 (sICAM-1) and E-selectin. Thalidomide is also a potent anti-angiogenic agent (D’Amato *et al*, 1994) due to suppression of VEGF and basic fibroblast growth factor (bFGF) secretion and inhibition of endothelial cell proliferation. VEGF receptor 2 (VEGFR2), principally expressed on the endothelial cell surface, mediates the pro-angiogenic response to VEGF (Shibuya, 2006). VEGFR2 is also found in a soluble truncated form of VEGF receptor-2 (sVEGFR2), which has been studied as a biomarker of tumour angiogenesis (Ebos *et al*, 2008). Significant decreases in sVEGFR2 plasma levels have been reported in clinical studies with anti-angiogenic therapy for NSCLC (Hanrahan *et al*, 2010; Nikolinakos *et al*, 2010), notably with tyrosine kinase inhibitors. It is not known whether the anti-angiogenic properties of thalidomide alter circulating sVEGFR2 levels.

Thalidomide combined with chemotherapy in SCLC was well tolerated in a single arm phase II study with a response rate of 17/25 (68%) (Lee *et al*, 2008). Thalidomide 200 mg once daily was therefore evaluated in combination with standard chemotherapy in two prospective phase III-randomised, double-blind, placebo-controlled trials in patients with SCLC (Lee *et al*, 2009b) and advanced NSCLC (Lee *et al*, 2009a) that enrolled a total of 1446 patients. Neither study showed that thalidomide in combination with chemotherapy increased overall survival (OS) compared with chemotherapy alone. However, a significant increase in thromboembolic events was seen in patients randomised to thalidomide.

Serial plasma samples were collected in an unselected cohort of patients enrolled in these two lung cancer studies. Here, we describe the analysis of circulating angiogenic biomarkers in this sample set. The objectives of this study were to determine the relationship between baseline angiogenic biomarkers, and other prognostic factors and outcome, to determine the interaction between angiogenic factors and treatment, and to investigate changes in angiogenic biomarkers due to thalidomide treatment.

## Patients and methods

### Patients and study design

The two multicenter, randomised, phase III clinical trials are described in detail elsewhere (Lee *et al*, 2009a, 2009b). Briefly, between May 2003 and September 2008, 724 patients with limited or extensive stage SCLC were treated with up to six cycles of carboplatin and etoposide chemotherapy every 3 weeks, and randomly assigned to receive either oral thalidomide or matching placebo for up to 2 years (LLCG study 12, ISRCTN16174527). A total of 722 patients with stage IIIB or stage IV NSCLC were treated with up to four cycles of gemcitabine and carboplatin chemotherapy every 3 weeks and randomly assigned to receive either oral thalidomide or matching placebo for up to 2 years (LLCG study 14, ISRCTN77341241). Both clinical studies were approved by the relevant ethics authorities and written informed consent was obtained from all patients. Plasma sample collection for biomarker analysis was planned from 120 patients (60 from each study) in selected participating centres. Plasma sample collection for the biomarker study was optional and only patients who consented to this are included in this analysis.

### Plasma sample collection and analyses

Plasma samples were prepared from venous blood samples collected before treatment cycles 1 (C1) and 4 (C4). Samples were collected in EDTA tubes, centrifuged at 2000 r.p.m. for 10 min and stored at −80°C until analysis. Samples were collected from 2003 to 2006 and stored at University College London until analysis at the University of Sheffield in 2011. Analysis was performed to measure levels of the angiogenic factors VEGF, sVEGFR-2, bFGF, TNF-*α*, IL-8 and sICAM-1, selected because of the known mechanism of thalidomide activity. During sample analysis, investigators were blinded to outcome and treatment group. Angiogenic factors were analysed by enzyme-linked immunosorbent assays (ELISA) (Quantikine, R&D Systems Europe Ltd, Abingdon, UK), as per the manufacturers’ instructions. A standard concentration curve was produced for each ELISA plate with the manufacturers’ control solution and used to calculate plasma concentrations. Serial samples were assessed on the same ELISA plate to reduce inter-experimental variability. Baseline levels and changes at C4 were correlated with stage, response rate and progression-free survival (PFS). Levels of angiogenic factors were compared between patients on thalidomide and those on placebo to investigate changes as a consequence of thalidomide therapy.

### Statistical methods

Logarithmic transformation to a normal distribution was performed prior to analysis of baseline VEGF, bFGF, TNF-*α*, IL-8 and sICAM-1. Baseline sVEGFR-2 and changes in angiogenic factor levels (from C1 to C4) were normally distributed without transformation. The independent samples *t*-test was used to assess differences between SCLC and NSCLC. The paired *t*-test was used to analyse changes in angiogenic factor levels from C1 to C4. The independent samples *t*-test was also used to analyse associations between angiogenic factor levels, tumour stage and best response. PFS was measured from the date of randomisation to the date of progression of disease, or of death from any cause. Differences in PFS were assessed using measurements of angiogenic factors expressed both as a continuous variable and dichotomised at the median to distinguish high and low groups. Cox-regression analysis was used to calculate hazard ratios (HR) for PFS, and multivariate analysis of survival was performed using the backward selection method. Statistical analysis was performed using SPSS 16.0 for Windows (IBM, Armonk, NY, USA).

## Results

### Patient population

In this biomarker study, 164 plasma samples were analysed from 95 patients. About 100% of patients contributed baseline (C1) samples and 73% (69/95) at C4. There were a similar number of patients with SCLC and NSCLC, and in the thalidomide and placebo groups. The patients’ characteristics are compared with those from the two parent clinical trials in Table 1. The distribution of age, gender and stage were similar to the clinical trial population but the biomarker study population was of slightly better performance status (not significant) and had a different pattern of NSCLC histological subtypes.

At the time of database lock, 87 of the 95 patients (92%) included in this biomarker study had progressed and 83/95 (87%) had died. As in the parent clinical trials, a comparison of the survival data for the biomarker study population showed no survival advantage for thalidomide compared with placebo in SCLC, NSCLC or the combined group. Median OS was 11.5 and 10.7 months for thalidomide and placebo groups, respectively, with an HR of 1.25 (95% CI 0.81–1.94; *P*=0.32). Median PFS was 6.8 and 7.3 months for thalidomide and placebo groups, respectively, with an HR of 1.24 (95% CI 0.80–1.90; *P*=0.36). There was also no significant difference in response rate by treatment group (partial response (PR): thalidomide (49%) *vs* placebo (36%); *P*=0.19).

In this study, earlier tumour stage was associated with a significantly improved PFS in SCLC, but not NSCLC. The HR for progression in SCLC with extensive disease (ED) was 2.70 (95% CI 1.40–5.21; *P*=0.003). In NSCLC, the HR for PFS with stage IV was 0.95 (95% CI 0.52–1.73; *P*=0.86). A trend towards decreased PFS with deteriorating WHO performance status was seen in SCLC with an HR 1.51 (95% CI 0.98–2.33; *P*=0.060), although in this study population the number of patients with poor performance status was small.

In the clinical trials, patients treated with thalidomide had an increased risk of a thrombotic event compared with patients receiving placebo. However, in this sub-study the difference was not significant (20% of patients developed thrombosis on thalidomide compared with 17% on placebo; *P*=0.89). The small sample size prohibited biomarker comparisons between thrombotic sub-groups.

### Analysis of baseline angiogenic biomarkers for pooled treatment groups

There were no statistically significant differences between patients with SCLC and NSCLC in angiogenic factors at baseline (C1) (Table 2). In SCLC, baseline VEGF and sICAM-1 were, on average, significantly lower in limited disease (LD) compared with ED, and there was a suggestion of lower IL-8 in LD (Table 3). There were no significant differences in baseline angiogenic factors between NSCLC stage IIIB and stage IV. There were no significant differences in baseline factors between patients who had a PR to treatment and those with stable (SD) or progressive disease (PD) for SCLC, NSCLC or the combined group (Table 4).

Survival analysis showed a significant increased risk of progression with high baseline IL-8 for SCLC and NSCLC patient groups combined, with an HR of 1.61 (95% CI 1.05–2.47; *P*=0.028) (Table 5). Subgroup survival analysis showed high baseline IL-8 was associated with a significantly worse prognosis in SCLC but not NSCLC, with an HR for progression of 1.94 (95% CI 1.05–3.59; *P*=0.036). Subgroup analysis also identified a significant relationship between PFS and baseline sICAM-1 in SCLC for pooled treatment arms but not in NSCLC, with an HR for progression with high baseline sICAM-1 in SCLC of 2.20 (95% CI 1.16–4.18; *P*=0.016). Similarly, there was a suggestion of an increased risk of progression with high baseline VEGF in SCLC, but not NSCLC; HR=1.79 (95% CI 0.97–3.30; *P*=0.063). No other significant differences were seen between PFS and baseline angiogenic factors in subgroup or pooled analysis, irrespective of the angiogenic factors being treated as categorical or continuous data.

In multivariate analysis of survival including baseline angiogenic factors, stage and performance status, stage was the only significant prognostic factor in SCLC.

### Changes in angiogenic biomarkers after chemotherapy for pooled treatment groups

**Figure 1:**
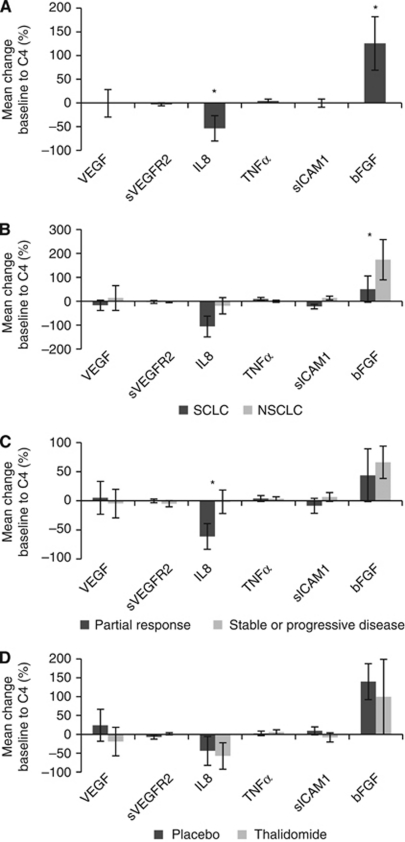
Changes in angiogenic biomarkers between baseline and C4. Each figure shows mean changes expressed as a percentage of baseline values. (**A**) Changes in angiogenic biomarkers for the combined data set. *P*-values were calculated to compare C1 with C4 values using the paired *t*-test. (**B**) Changes in angiogenic biomarkers for SCLC and NSCLC. *P*-values were calculated using the independent *t*-test. (**C**) Changes in angiogenic biomarkers according to best tumour response. *P*-values were calculated using the independent *t*-test. (**D**) Changes in angiogenic biomarkers for thalidomide and placebo groups. *P*-values were calculated using the independent *t*-test; none were significant. ^*^P<0.05, error bars represent 1 standard error of the mean.

In the combined SCLC and NSCLC data set, there were significant changes in IL-8 and bFGF levels between C1 and C4 (Figure 1A). There was a decrease of IL-8 14.6 pg ml^−1^ (±7.3; *P*=0.049) and bFGF increased by 14.5 pg ml^−1^ (±6.5; *P*=0.029). Only sICAM-1 demonstrated a significant difference between SCLC and NSCLC in mean change in factor level. In SCLC, sICAM-1 reduced by 70.4 ng ml^−1^ (±51.0) whereas in NSCLC sICAM-1 increased by 60.7 ng ml^−1^ (±34.0) (*P*=0.033). Changes in other investigated angiogenic factors did not differ significantly between SCLC and NSCLC (Figure 1B).

Reduction in IL-8 for pooled treatment arms was associated with response in both SCLC and NSCLC. Patients with a PR had a larger decrease in IL-8 from C1 to C4 compared with those with SD and PD. Mean decrease in IL-8 from patients with PR was 31.4 pg ml^−1^ (±11.3) compared with 0.8 pg ml^−1^ (±8.9) with SD and PD (*P*=0.035). There was a suggestion of a trend on further separating SD −1.5 pg ml^−1^ (±10.8) and PD +2.1 pg ml^−1^ (±13.8), (one-way ANOVA, *P*=0.109), but the number of patients with PD were small (*n*=8). Changes in the levels of other angiogenic factors for pooled treatment arms did not predict tumour response in SCLC, NSCLC or the combined histology group (Figure 1C). Furthermore, there were no significant differences in PFS by change in angiogenic factors (Table 5).

### Angiogenic factors and thalidomide

Biomarker levels were compared at C1 to adjust for differences between treatment groups at baseline. Only baseline sVEGFR-2 was significantly different between the two treatment groups (mean sVEGFR-2 at C1 in the thalidomide arm was 7080.7 pg ml^−1^ (±2410.3) compared with 8505.0 pg ml^−1^ (±2833.2) in the placebo arm (*P*=0.010)). No significant differences were seen in the mean change in angiogenic factor levels from C1 to C4 between thalidomide and placebo groups (Figure 1D).

Subgroup analysis was performed to explore whether baseline levels of angiogenic biomarkers predicted response to thalidomide. For patients who received thalidomide, there were no significant differences in tumour response or PFS between those with high baseline levels compared with those with low baseline levels (Table 6). Analysis to quantify the relationship (i.e., test for interaction) between thalidomide and biomarker was therefore not supported.

Finally, the parent clinical trial of thalidomide in NSCLC suggested a potential survival benefit for thalidomide in patients with squamous histology. Biomarker analysis was limited because the number of patients with squamous NSCLC was small (*n*=11). Compared with other NSCLC histology patients, those with squamous disease had a significantly higher bFGF at baseline (median bFGF with squamous histology was 31.5 pg  ml^−1^ (range 3.3–143.1) compared with 6.9 pg ml^−1^ (range 0.0–112.5) with other NSCLC histology (*P*=0.022)), and a suggestion of a higher VEGF (median VEGF with squamous histology was 265.7 pg ml^−1^ (range 50.2–1360.8) compared with 141.2 pg ml^−1^ (range 31.3–761.9) with other NSCLC histology (*P*=0.07)). There were no significant differences in change in angiogenic factor levels from C1 to C4 between squamous and other NSCLC histology.

## Discussion

Thalidomide is a drug with multiple mechanisms of action including inhibition of angiogenesis. Treatment with thalidomide failed to provide any survival benefit in two large phase III trials in SCLC and NSCLC (Lee *et al*, 2009a, 2009b). We studied pre-treatment and pre-cycle four plasma levels of VEGF, sVEGFR-2, IL-8, TNF-*α*, sICAM-1 and bFGF in an unselected cohort of patients from these trials to analyse the biological effects of thalidomide treatment, and to investigate their utility as biomarkers in lung cancer. The biomarker study population was broadly representative of the clinical trial population, although only 6.6% of patients recruited to the two clinical lung cancer studies had samples collected for analysis of angiogenic growth factors; and this is an important limitation of our study.

The biology of SCLC and NSCLC is different, and in our analysis we considered these groups separately. However, analysis of the combined data set was also justified. Differences between SCLC and NSCLC at baseline (C1) and in the change to angiogenic factor levels from C1 to C4 (excluding sICAM-1) were not statistically significant. Furthermore, an important aim of this study was to investigate changes in angiogenic factors as a consequence of thalidomide treatment, and it was predicted these changes would be similar in both SCLC and NSCLC patients.

A wide range of values was seen in the levels of angiogenic biomarkers quantified in this study, similar to that reported in other biomarker studies in lung cancer (Dowlati *et al*, 2008; Hanrahan *et al*, 2010). Low baseline levels of IL-8, VEGF and sICAM-1 were associated with limited stage SCLC. These factors were also associated with an improved PFS, although in multivariate analysis only tumour stage was significant. Comparisons between baseline angiogenic factors in stage IIIB and stage IV NSCLC were not significant, which is unsurprising as early stage NSCLC were excluded from the clinical trial. Changes from C1 to C4 were seen in IL-8 and sICAM-1 levels, and changes in IL-8 were associated with tumour response in the combined NSCLC and SCLC data set.

An important effect of thalidomide is to decrease TNF-*α* through degradation of its mRNA (Melchert and List, 2007). However, in this study there was no difference in TNF-*α* at C4 between those on thalidomide and those on placebo. Overall TNF-*α* levels increased on study and increased more in patients on thalidomide than in those on placebo, although this difference was not significant. TNF-*α* expression did not correlate with the other investigated angiogenic factors; however, the anti-angiogenic activity of thalidomide is independent of its TNF-*α* effect (Dredge *et al*, 2002) and is mediated through reduced secretion of angiogenic growth factors including VEGF and inhibition of endothelial cell proliferation (Melchert and List, 2007). We found no significant differences in the change to angiogenic biomarker levels from C1 to C4 between thalidomide and placebo groups; furthermore, high baseline levels of angiogenic factors were not associated with response to thalidomide. We conclude there were no detectable effects on angiogenic factor levels as a result of thalidomide therapy in this study. Published data on the effects of thalidomide therapy on circulating angiogenic biomarkers is conflicting. Phase II studies of thalidomide in multiple myeloma have concluded that a higher baseline VEGF levels were associated with response to treatment ((Mileshkin *et al*, 2007), and clinical responses associated with decreases in VEGF and bFGF levels (Bertolini *et al*, 2001). Other studies of thalidomide in multiple myeloma have observed no change (Thompson *et al*, 2003), or even increase in VEGF and bFGF levels with treatment (Hatjiharissi *et al*, 2004). Two parallel non-randomised phase II studies in patients with malignant mesothelioma treated with single agent thalidomide or thalidomide combined with carboplatin and gemcitabine reported pre-treatment VEGF serum levels were prognostic, and increases in VEGF levels on treatment were associated with a worse prognosis (Kao *et al*, 2012). A small phase II trial of neo-adjuvant carboplatin–gemcitabine chemotherapy with thalidomide in 15 patients with stage IIB–IIIA NSCLC showed that a higher baseline IL-8 was associated with a significantly greater risk of disease recurrence post-operatively, although an increase in IL-8 after treatment was associated with a reduced risk of recurrence (Dudek *et al*, 2009). Biomarker levels in this study of patients with relatively low volume disease were lower than in our NSCLC patients with stage IIIB and IV disease.

IL-8 emerged as a factor of interest in our analysis. Through the G protein-coupled receptors CXCR1 and CXCR2, it exerts both inflammatory and angiogenic responses, and can directly stimulate cancer cell proliferation and survival (Waugh and Wilson, 2008). The expression of IL-8 and its receptors has been catalogued in a panel of SCLC and NSCLC cell lines, and was identified as an autocrine and/or paracrine growth factor in these cells (Zhu *et al*, 2004). In a study of resected NSCLC, IL-8 protein expression predominated in tumour cells and to a lesser degree in tumour-associated macrophages. Expression of IL-8 mRNA correlated with tumour micro-vessel density, and increased expression was significantly associated with more advanced stage disease, earlier recurrence and reduced OS (Yuan *et al*, 2000). Increases in serum IL-8 on treatment were associated with a reduced PFS in a recent study of vandetanib and chemotherapy in NSCLC (Hanrahan *et al*, 2010); however, a small study in SCLC identified no relationship with serum IL-8 and tumour stage, chemotherapy response or PFS (Tas *et al*, 2006). Further studies are required to clarify the relationship between IL-8 and tumour burden in lung cancer, and how levels change with systemic therapy.

In this study, the interval between plasma samples was relatively long (9 weeks). A study of the anti-angiogenic tyrosine kinase inhibitor vandetanib in NSCLC demonstrated that changes in biomarker levels occurred early in treatment (day 8) and were later lost in the noise of chemotherapy-induced changes (Hanrahan *et al*, 2010). Thus, early changes in angiogenic biomarkers could have been missed in our study. The absence of detectable changes in angiogenic factors with thalidomide therapy seen in this study, and lack of therapeutic benefit identified in the clinical lung cancer studies might have been due to an inadequate thalidomide dose, although this dose was sufficient to significantly increase thromboembolic events. A phase III study comparing 400 mg daily of thalidomide with placebo in patients with chemo-responsive SCLC, however, also failed to demonstrate a significant improvement in survival, but was associated with an increased toxicity (Pujol *et al*, 2007).

The response to angiogenesis-targeted therapy in lung cancer clinical trials has thus far proved disappointing (Ulahannan and Brahmer, 2011). A predictive biomarker is required to target the subpopulation of patients who can benefit from these agents. Further studies are required to investigate the direct biological effects of these drugs on tumours in patients. Circulating biomarkers are a surrogate measure of these changes but their collection and analysis is both more feasible and practical, and it is hoped it will enable identification of patient sub-populations sensitivity to therapy (Reinmuth *et al*, 2010). In our analysis of an unselected cohort of patients from two lung cancer studies of chemotherapy with or without thalidomide, significant changes were seen in angiogenic biomarker levels in association with treatment. However, changes in factor levels could not be attributed to thalidomide therapy, and elevated baseline angiogenic factors did not predict response to thalidomide. The use of IL-8 as a biomarker in lung cancer requires validation in larger studies.

## Figures and Tables

**Table 1:** Patient characteristics of the biomarker population, shown by tumour type and the parent clinical population

	**SCLC**		**NSCLC**		**SCLC and NSCLC combined**		**Parent studies combined**	
	***n***	**%**	***N***	**%**	***n***	**%**	***N***	**%**
Total	47	100	48	100	95	100	1446	100
								
*Treatment*								
Thalidomide	25	53	24	50	49	52	737	51
Placebo	22	47	24	50	46	48	709	49
								
*Age at random assignment*								
⩾50	45	96	41	85	86	91	1342	93
								
*Gender*								
Male	29	62	34	71	63	66	877	61
Female	18	38	14	29	32	34	569	39
								
*Stage*								
Limited stage/stage IIIb	20	43	22	46	42	44	690	48
Extensive stage/stage IV	27	57	26	54	53	56	756	52
								
*WHO performance status*								
0	13	28	16	33	29	31	344	24
1	24	51	32	67	56	59	833	57
2	8	17	0	0	8	8	227	16
3	2	4	0	0	2	2	42	3
								
*Cell type (NSCLC only)*								
Squamous cell			11	23			239	33
Adenocarcinoma			17	35			268	37
Large cell			6	13			47	7
Other			14	29			168	23

Abbreviations: NSCLC=non-small cell lung cancer; SCLC=small cell lung cancer.

**Table 2:** Angiogenic biomarker levels at baseline (C1) and pre-cycle 4 (C4) for SCLC and NSCLC

		**SCLC**			**NSCLC**			
**Angiogenic factor**	**Time**	***n***	**Median (pg ml^−1^)**	**Range (pg ml^−1^)**	***n***	**Median (pg ml^−1^)**	**Range (pg ml^−1^)**	***P***
VEGF	C1	47	144.6	23.2–912.0	48	149.6	10.3–1360.8	0.58
	C4	33	145.5	22.4–1055.1	36	129.9	21.6–3051.9	0.93
sVEGFR-2	C1	47	7833.8	90.0–13711.0	48	8243.0	3302.0–14 515.0	0.15
	C4	33	8061.9	530.0–7092.2	36	7400.0	3780.0–14 336.3	0.35
IL-8	C1	47	23.0	0.0–335.8	48	30.7	5.8–198.0	0.08
	C4	33	15.2	3.5–104.3	36	22.4	1.6–240.0	0.25
TNF-*α*	C1	44	23.7	7.8–63.5	46	31.4	0.0–121.0	0.97
	C4	30	22.6	11.4–60.0	35	28.4	0.0–112.3	0.98
sICAM-1^a^	C1	46	321.1	160.3–1307.4	47	436.6	132.9–933.2	0.11
	C4	31	326.8	195.8–804.6	35	434.3	165.0–1127.1	**0.039**
bFGF	C1	46	12.0	0.6–181.3	46	12.6	0.0–143.1	0.30
	C4	31	11.9	0.0–176.0	35	17.1	0.0–383.4	0.94

Abbreviations: bFGF=basic fibroblast growth factor; IL-8=interleukin-8; NSCLC=non-small cell lung cancer; SCLC=small cell lung cancer; sICAM-1=soluble intercellular adhesion molecule-1; sVEGFR-2=soluble truncated form of vascular endothelial growth factor receptor-2; TNF-*α=*tumour necrosis factor-*α*; VEGF=vascular endothelial growth factor.

The *P*-value was calculated using the independent *t*-test, comparing the mean values for SCLC with NSCLC, following logarithmic transformation of the data to achieve a normal distribution where necessary. *P*-values <0.05 are highlighted in bold.

a sICAM-1 was measured in ng ml^−1^.

**Table 3:** Comparison of SCLC and NSCLC baseline factors by tumour stage

		**SCLC**			**NSCLC**		
**Angiogenic factor**	**Stage**	**Median**	**Range**	***P***	**Median**	**Range**	***P***
VEGF	LD/IIIB	121.8	23.2–308.1	**0.046**	156.5	31.3–952.9	0.77
	ED/IV	151.3	27.0–912.0		146.3	59.2–1360.8	
sVEGFR-2	LD/IIIB	7892.5	507.5–13711.0	0.95	8372.0	3302.0–11 760.0	0.96
	ED/IV	7580.0	90.0–11346.3		7317.1	4271.0–14 515.0	
IL-8	LD/IIIB	12.1	0.0–335.8	0.07	24.2	8.3–198.0	0.93
	ED/IV	24.5	1.8–244.8		32.3	8.2–146.7	
TNF-*α*	LD/IIIB	22.9	9.1–63.5	0.68	38.6	2.2–121.0	0.58
	ED/IV	24.3	7.8–43.7		24.8	0.0–96.1	
sICAM-1^a^	LD/IIIB	305.5	168.1–508.6	**0.008**	383.3	160.7–792.4	0.31
	ED/IV	388.4	160.3–1307.4		443.3	132.9–933.2	
bFGF	LD/IIIB	9.1	0.6–73.0	0.13	7.1	0.0–143.1	0.77
	ED/IV	19.7	1.0–181.3		12.6	0.0–112.5	

Abbreviations: bFGF=basic fibroblast growth factor; ED=extensive disease; IL-8=interleukin-8; LD=limited disease; NSCLC=non-small cell lung cancer; SCLC=small cell lung cancer; sICAM-1=soluble intercellular adhesion molecule-1; sVEGFR-2=soluble truncated form of vascular endothelial growth factor receptor-2; TNF-*α=*tumour necrosis factor-*α*; VEGF=vascular endothelial growth factor.

*P*-values were calculated using the independent *t*-test, following logarithmic transformation where necessary. *P*-values <0.05 are highlighted in bold.

a sICAM-1 measured in ng ml^−1^.

**Table 4:** Baseline levels (C1) of angiogenic factors according to best response

**Angiogenic factor**	**Best response**	**Median values at C1 in pg ml^−1^ (range)**	***P***
VEGF	PR	155.1 (23.2–1360.8)	0.84
	SD and PD	138.6 (10.3–1360.8)	
sVEGFR2	PR	8049.4 (90.0–11 892.5)	0.41
	SD and PD	7936.4 (656.3–14 515.0)	
IL-8	PR	23.2 (0.0–335.8)	0.57
	SD and PD	30.7 (2.9–174.6)	
TNF*α*	PR	26.3 (2.2–121.0)	0.26
	SD and PD	25.5 (0.0–87.4)	
sICAM1^a^	PR	354.5 (160.3–1307.4)	0.81
	SD and PD	377.6 (132.9–1056.7)	
bFGF	PR	10.3 (0.0–143.11)	0.67
	SD and PD	13.9 (0.0–181.3)	

Abbreviations: bFGF=basic fibroblast growth factor; IL-8=interleukin-8; NSCLC=non-small cell lung cancer; PD=progressive disease; PR=partial response; SCLC=small cell lung cancer; SD=stable disease; sICAM-1=soluble intercellular adhesion molecule-1; sVEGFR-2=soluble truncated form of vascular endothelial growth factor receptor-2; TNF-*α=*tumour necrosis factor-*α*; VEGF=vascular endothelial growth factor.

*P*-values were calculated using the independent *t*-test and confirmed using logistic regression.

a sICAM-1 measured in ng ml^−1^.

**Table 5:** Angiogenic biomarker effects on PFS for SCLC and NSCLC patients combined

**Angiogenic factor**	**Hazard ratio (95% CI) by angiogenic factor at baseline**	***P***	**Hazard ratio (95% CI) by change in factor from C1 to C4**	***P***
VEGF	1.17 (0.76–1.78)	0.48	0.80 (0.49–1.31)	0.38
sVEGFR2	1.07 (0.70–1.64)	0.75	1.24 (0.76–2.03)	0.39
IL-8	1.61 (1.05–2.47)	**0.028**	0.78 (0.48–1.29)	0.33
TNF*α*	0.77 (0.50–1.20)	0.25	1.42 (0.85–2.36)	0.19
sICAM1	1.35 (0.87–2.07)	0.18	0.92 (0.55–1.52)	0.73
bFGF	1.04 (0.67–1.60)	0.88	0.89 (0.53–1.48)	0.65

Abbreviations: bFGF=basic fibroblast growth factor; CI=confidence interval; IL-8=interleukin-8; NSCLC=non-small cell lung cancer; PFS=progression-free survival; SCLC=small cell lung cancer; sICAM-1=soluble intercellular adhesion molecule-1; sVEGFR-2=soluble truncated form of vascular endothelial growth factor receptor-2; TNF-*α=*tumour necrosis factor-*α*; VEGF=vascular endothelial growth factor.

Factors were dichotomised around the median value into high and low groups. *P*-values <0.05 are highlighted in bold.

**Table 6:** Angiogenic biomarker effects on PFS for thalidomide

**Angiogenic factor**	**Hazard ratio (95% CI) by angiogenic factor at baseline**	***P***
VEGF	0.89 (0.49–1.59)	0.67
sVEGFR2	1.49 (0.82–2.71)	0.20
IL-8	1.33 (0.75–2.38)	0.33
TNF*α*	0.95 (0.51–1.74)	0.86
sICAM1	0.82 (0.44–1.51)	0.52
bFGF	1.00 (0.55–1.81)	0.99

Abbreviations: bFGF=basic fibroblast growth factor; CI=confidence interval; IL-8=interleukin-8; PFS=progression-free survival; sICAM-1=soluble intercellular adhesion molecule-1; sVEGFR-2=soluble truncated form of vascular endothelial growth factor receptor-2; TNF-*α=*tumour necrosis factor-*α*; VEGF=vascular endothelial growth factor.

Factors were dichotomised around the median value into high and low groups.
